# Analysis of newly detected tetracycline resistance genes and their flanking sequences in human intestinal bifidobacteria

**DOI:** 10.1038/s41598-017-06595-0

**Published:** 2017-07-24

**Authors:** Na Wang, Xiaomin Hang, Min Zhang, Xianglong Liu, Hong Yang

**Affiliations:** 1 State Key Laboratory of Microbial metabolism, and School of Life Science & Biotechnology, Shanghai Jiao Tong University, Shanghai, 200240 P.R. China; 2 Institute of Bio-medicine, Shanghai Jiao Da Onlly Company Limited, Shanghai, 200233 P. R. China

## Abstract

Due to tetracycline abuse, the safe bifidobacteria in the human gastrointestinal intestinal tract (GIT) may serve as a reservoir of tetracycline resistance genes. In the present investigation of 92 bifidobacterial strains originating from the human GIT, tetracycline resistance in 29 strains was mediated by the *tet*(W), *tet*(O) or *tet*(S) gene, and this is the first report of *tet*(O)- and *tet*(S)-mediated tetracycline resistance in bifidobacteria. Antibiotic resistance genes harbored by bifidobacteria are transferred from other bacteria. However, the characteristics of the spread and integration of tetracycline resistance genes into the human intestinal bifidobacteria chromosome are poorly understood. Here, conserved sequences were identified in bifidobacterial strains positive for *tet*(W), *tet*(O), or *tet*(S), including the *tet*(W), *tet*(O), or *tet*(S) and their partial flanking sequences, which exhibited identity with the sequences in multiple human intestinal pathogens, and genes encoding 23 S rRNA, an ATP transporter, a Cpp protein, and a membrane-spanning protein were flanking by the 1920-bp *tet*(W), 1920-bp *tet*(O), 1800-bp *tet*(O) and 252-bp *tet*(S) in bifidobacteria, respectively. These findings suggest that tetracycline resistance genes harbored by human intestinal bifidobacteria might initially be transferred from pathogens and that each kind of tetracycline resistance gene might tend to insert in the vicinity of specific bifidobacteria genes.

## Introduction

There are up to 10^13^–10^14^ total bacteria in the human gastrointestinal intestinal tract (GIT)^1, 2^. Due to the abuse of tetracycline in the clinical and nonclinical treatment of various human infections^3^, the carriage of tetracycline resistance genes by bacteria in the human GIT has been an area of intense investigation^4^. Most studies have focused on the tetracycline resistance genes carried by clinical pathogens or opportunistic pathogens^5^ and have continuously detected new tetracycline resistance genes harbored by the intestinal pathogens, such as the *tet*(40) gene in the human intestinal firmicute bacterium^6^. However, because bifidobacteria are ingested as probiotics in the human GIT and have acquired a “generally regarded as safe” (GRAS) status^7–9^, so far, only *tet*(W)- and *tet*(M)-mediated tetracycline resistance have been detected in intestinal bifidobacteria of human origin^10–13^, and only *tet*(L)-, *tet*(O/W)-, *tet*(W/32/O)-, and *tet*(O/W/32/O/W/O)-mediated tetracycline resistance have been detected in intestinal bifidobacteria of pig origin^14^. Therefore, it remains unknown whether tetracycline resistance genes other than *tet*(W) and *tet*(M) can be detected in the bifidobacterial strains originating in the human GIT.

Antibiotic resistance (AR) genes within potentially mobile elements can spread horizontally across genera in the human GIT^15^. Comparative analysis of sequences flanking the same AR gene in one genus of bacteria can therefore further reveal the spread characteristics of the AR gene. However, although two tetracycline resistance genes [*tet*(W) and *tet*(M)] have been detected in human intestinal bifidobacteria^10–13^, only the sequences flanking the *tet*(W) gene in bifidobacteria have been analyzed^10, 12^. Scott previously found a conserved *tet*(W) gene sequence of 2154 bp in 10 gut bifidobacterial strains of 5 species^12^. Ammor analyzed the flanking sequences of the *tet*(W) genes in another six human intestinal bifidobacteria and found an *orf*Y gene in the downstream flanking region of the *tet*(W) gene in one *B*. *thermophilum* strain and one *B*. *longum* strain and a transposase gene in the downstream flanking region of the *tet*(W) gene in two *B*. *longum* strains^10^. Based on these results, it is not possible to determine whether the *tet*(W) gene inserts into common sites in the chromosome of the human intestinal bifidobacteria or whether other tetracycline resistance genes may exhibit conservation in their integration into the human intestinal bifidobacteria chromosome.

As a result of the misuse and overuse of tetracycline, the traditionally safe bifidobacteria in the human GIT may serve as a reservoir of tetracycline resistance genes and increasingly become a threat to human health. Therefore, this study was performed to assess 92 bifidobacterial strains isolated from the feces of 14 healthy individuals, one type strain and seven commercial strains via phenotypically and genotypically screening the acquired tetracycline resistance profiles and to comparatively analyze the upstream and downstream sequences flanking the tetracycline resistance genes harbored by different strains.

## Results

### Tetracycline susceptibility profiles

The MIC values of tetracycline in the 100 bifidobacterial strains tested are presented in Tables 1 and 2. Twenty-nine bifidobacterial strains, including the seven *Bifidobacterium longum* strains shown in Table 1 and two *Bifidobacterium bifidum* strains, six *Bifidobacterium pseudocatenulatum* strains, 13 *Bifidobacterium lactis* strains and one *Bifidobacterium breve* strain shown in Table 2, exhibited strong tetracycline resistance [minimum inhibitory concentration (MIC) ≥256 μg/ml], with MIC values that higher than the breakpoint for *Bifidobacterium* defined by the European Food Safety Authority (EFSA) (MIC = 8 μg/ml)^16^.

**Table 1:** MIC susceptibility profiles of tetracycline and the corresponding genotypes for 45 *B*. *longum* strains one *B*. *infantis* strain.

Species	Strain	Origin	MIC (μg/ml)	Tetracycline resistance genes			
				*tet*(W)	*tet*(O)	*tet*(S)	The other 10 genes
*B*. *infantis*	Pronova BI211^a^	Human	<0.016	−	−	−	−
*B*. *longum*	Pronova BL88-Onlly^a^	Human	<0.016	−	−	−	−
	A33	Child feces	<0.016	−	−	−	−
	A42	Child feces	<0.016	−	−	−	−
	W11	Adult feces	<0.016	−	−	−	−
	W12	Adult feces	<0.016	−	−	−	−
	W14	Adult feces	<0.016	−	−	−	−
	W210	Adult feces	<0.016	−	−	−	−
	W22	Adult feces	<0.016	−	−	−	−
	N34	Adult feces	<0.016	−	−	−	−
	N45	Adult feces	<0.016	−	−	−	−
	N51	Adult feces	<0.016	−	−	−	−
	Y27	Adult feces	<0.016	−	−	−	−
	Y35	Adult feces	<0.016	−	−	−	−
	Z21	Child feces	<0.016	−	−	−	−
	Z31	Child feces	<0.016	−	−	−	−
	D41	Child feces	<0.016	−	−	−	−
	D510	Child feces	<0.016	−	−	−	−
	D512	Child feces	<0.016	−	−	−	−
	D514	Child feces	<0.016	−	−	−	−
	X41	Child feces	<0.016	−	−	−	−
	H1	Child feces	<0.016	−	−	−	−
	H32	Child feces	<0.016	−	−	−	−
	L2	Adult feces	<0.016	−	−	−	−
	L8	Adult feces	<0.016	−	−	−	−
	N7	Adult feces	<0.016	−	−	−	−
	W211	Adult feces	<0.016	−	−	−	−
	W21	Adult feces	<0.016	−	−	−	−
	W24	Adult feces	<0.016	−	−	−	−
	W29	Adult feces	<0.016	−	−	−	−
	W212	Adult feces	<0.016	−	−	−	−
	W41	Adult feces	<0.016	−	−	−	−
	a44	Child feces	<0.016	−	−	−	−
	A31	Child feces	<0.016	−	−	−	−
	A44	Child feces	<0.016	−	−	−	−
	A45	Child feces	<0.016	−	−	−	−
	A47	Child feces	<0.016	−	−	−	−
	F7	Adult feces	<0.016	−	−	−	−
	Y2	Adult feces	<0.016	−	−	−	−
	H21	Child feces	≥256	−	+	−	−
	H34	Child feces	≥256	−	+	−	−
	F313	Adult feces	≥256	−	+	−	−
	F21	Adult feces	≥256	+	−	−	−
	X33	Child feces	≥256	+	−	−	−
	Y33	Adult feces	≥256	−	+	−	−
	Z1	Child feces	≥256	−	+	−	−

^a^Commercial strain obtained from the Shanghai Jiao Da Onlly Co. (Shanghai, PR China).

**Table 2:** MIC susceptibility profiles of tetracycline and the corresponding genotypes for 2 *B*. *adolescentis* strains, 3 *B*. *bifidum* strains, 12 *B*. *pseudocatenulatum* strains, 18 *B*. *breve* strains and 19 *B*. *lactis* strains.

Species	Strain	Origin	MIC (μg/ml)	Tetracycline resistance genes			
				*tet*(W)	*tet*(O)	*tet*(S)	The other 10 genes
*B*. *adolescentis*	W25	Adult feces	<0.016	−	−	−	−
	W42	Adult feces	<0.016	−	−	−	−
*B*. *bifidum*	Pronova BB47^a^	Human	<0.016	−	−	−	−
	Y24	Adult feces	≥256	+	−	−	−
	Y21	Adult feces	≥256	+	−	−	−
*B*. *pseudocatenulatum*	L37	Adult feces	<0.016	−	−	−	−
	W13	Adult feces	<0.016	−	−	−	−
	W28	Adult feces	<0.016	−	−	−	−
	N2	Adult feces	<0.016	−	−	−	−
	A35	Child feces	<0.016	−	−	−	−
	D52	Child feces	<0.016	−	−	−	−
	J56	Adult feces	≥256	+	−	−	−
	H23	Child feces	≥256	+	−	−	−
	Z25	Child feces	≥256	+	−	−	−
	a39	Child feces	≥256	+	−	−	−
	Y1	Adult feces	≥256	−	+		−
	F312	Adult feces	≥256	−	−	+	−
*B*. *breve*	ATCC 15700^b^	Human	<0.016	−	−	−	−
	Pronova BB8^a^	Human	<0.016	−	−	−	−
	BBW	Child feces	<0.016	−	−	−	−
	BBM	Child feces	<0.016	−	−	−	−
	BB2	Child feces	<0.016	−	−	−	−
	BB	Child feces	<0.016	−	−	−	−
	N1	Adult feces	<0.016	−	−	−	−
	N24	Adult feces	<0.016	−	−	−	−
	L211	Adult feces	<0.016	−	−	−	−
	W46	Adult feces	<0.016	−	−	−	−
	SQS3-56	Child feces	<0.016	−	−	−	−
	SQS3-64	Child feces	<0.016	−	−	−	−
	SQS5-51	Child feces	<0.016	−	−	−	−
	SQS5-52	Child feces	<0.016	−	−	−	−
	A34	Child feces	<0.016	−	−	−	−
	a313	Child feces	<0.016	−	−	−	−
	a37	Child feces	<0.016	−	−	−	−
	A27	Child feces	≥256	−	−	+	−
*B*. *lactis*	Pronova BL99^a^	Human	<0.016	−	−	−	−
	Pronova BL25^a^	Human	<0.016	−	−	−	−
	Pronova BI516^a^	Human	<0.016	−	−	−	−
	J316	Adult feces	<0.016	−	−	−	−
	F5	Adult feces	<0.016	−	−	−	−
	F18	Adult feces	<0.016	−	−	−	−
	F9	Adult feces	≥256	+	−	−	−
	F10	Adult feces	≥256	+	−	−	−
	F11	Adult feces	≥256	+	−	−	−
	F12	Adult feces	≥256	+	−	−	−
	J310	Adult feces	≥256	+	−	−	−
	J311	Adult feces	≥256	+	−	−	−
	J317	Adult feces	≥256	+	−	−	−
	L35	Adult feces	≥256	+	−	−	−
	L36	Adult feces	≥256	+	−	−	−
	L38	Adult feces	≥256	+	−	−	−
	L310	Adult feces	≥256	+	−	−	−
	L311	Adult feces	≥256	+	−	−	−
	L312	Adult feces	≥256	+	−	−	−

^a^Commercial strain obtained from the Shanghai Jiao Da Onlly Co. (Shanghai, PR China).

^b^Type strain.

### Detection of tetracycline resistance genes

As Tables 1 and 2 show, each of the 29 tetracycline-resistant bifidobacterial strains possessed one tetracycline resistance determinant [*tet*(W), or *tet*(O), or *tet*(S) gene], and none of the 13 tetracycline resistance determinants tested were detected in the 71 tetracycline-sensitive bifidobacterial strains. The occurrence of the *tet*(W), *tet*(O), and *tet*(S) genes among the 100 bifidobacterial strains of the seven *Bifidobacterium* species tested are further summarized in Table 3.

**Table 3:** Tetracycline resistance and occurrence of tetracycline resistance genes among 100 bifidobacterial strains of seven species.

Species	Total strain number	Tetracycline resistant strains	*tet*(W)	*tet*(O)	*tet*(S)
*B*. *adolescentis*	2	0	−	−	−
*B*. *infantis*	1	0	−	−	−
*B*. *longum*	45	7	2	5	−
*B*. *lactis*	19	13	13	−	−
*B*. *pseudocatenulatum*	12	6	4	1	1
*B*. *breve*	18	1	−	−	1
*B*. *bifidum*	3	2	2	−	−
Total	100	29	21	6	2

In the 21 *tet*(W)-positive strains, including 2 *B*. *longum subsp*. *longum* strains, 13 *B*. *animalis subsp*. *lactis* strains, 4 *B*. *pseudocatenulatum* strains, and 2 *B*. *bifidum* strains, *tet*(W) exhibited an identical DNA sequence of 1560 bp, which encoded a protein consisting of 520 amino acids that displayed 100% identity with the ribosomal protection protein tetW previously identified in *Bifidobacterium animalis subsp*. *lactis* strain IPLAIC4 (GenBank accession number GU361625.1).

In the 6 *tet*(O)-positive strains including 5 *B*. *longum subsp*. *longum* strains and one *B*. *pseudocatenulatum* strain, *tet*(O) exhibited an identical DNA sequence of 1457 bp, which encoded a protein consisting of 458 amino acids that displayed 100% identity with the ribosomal protection protein tetO previously identified in *Streptococcus suis* BM407 (GenBank FM252032.1).

In the two *tet*(S)-positive strains, *B*. *pseudocatenulatum* strain F312 and *B*. *breve* strain A27, *tet*(S) exhibited an identical DNA sequence of 210 bp, which encoded a protein consisting of 70 amino acids that displayed 100% identity with the ribosomal protection protein tetS previously identified in *Lactococcus lactis subsp*. *lactis* strain ILIBB-JZK (GenBank KF278750.1).

The complete sequence lengths of the *tet*(W), *tet*(S), and *tet*(O) genes were further confirmed by determining the sequences flanking the *tet*(W), *tet*(O), and *tet*(S) genes (see section “Sequence conservation of the *tet*(W), *tet*(O), *tet*(S) genes and their flanking regions”).

### Sequence conservation of the tet(W), tet(O), tet(S) genes and their flanking regions

**Figure 1:**
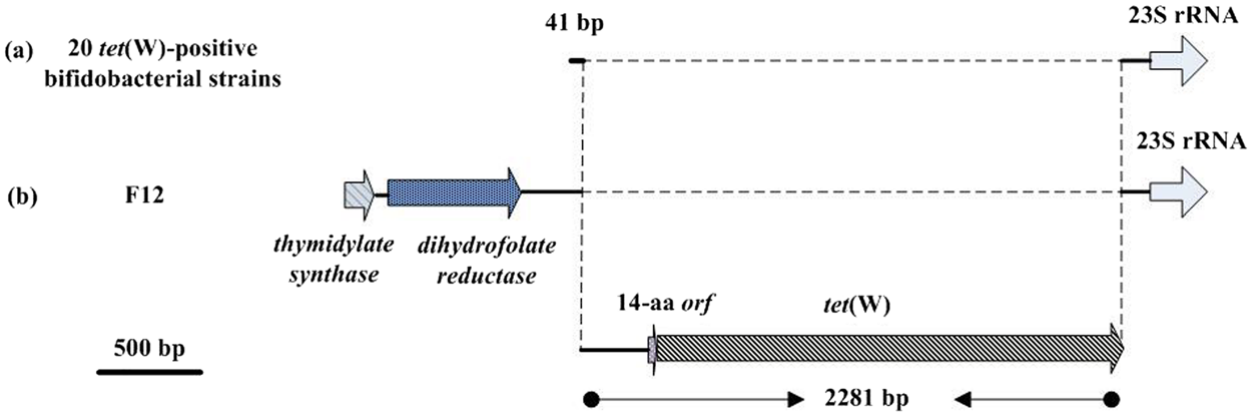
Genetic organization of the regions upstream and downstream of *tet*(W) in the 21 *tet*(W)-positive bifidobacterial strains. (**a**) 20 *tet*(W)-positive bifidobacterial strains. (**b**) The *tet*(W)-positive *B*. *animalis subsp*. *lactis* strain F12.

**Figure 2:**
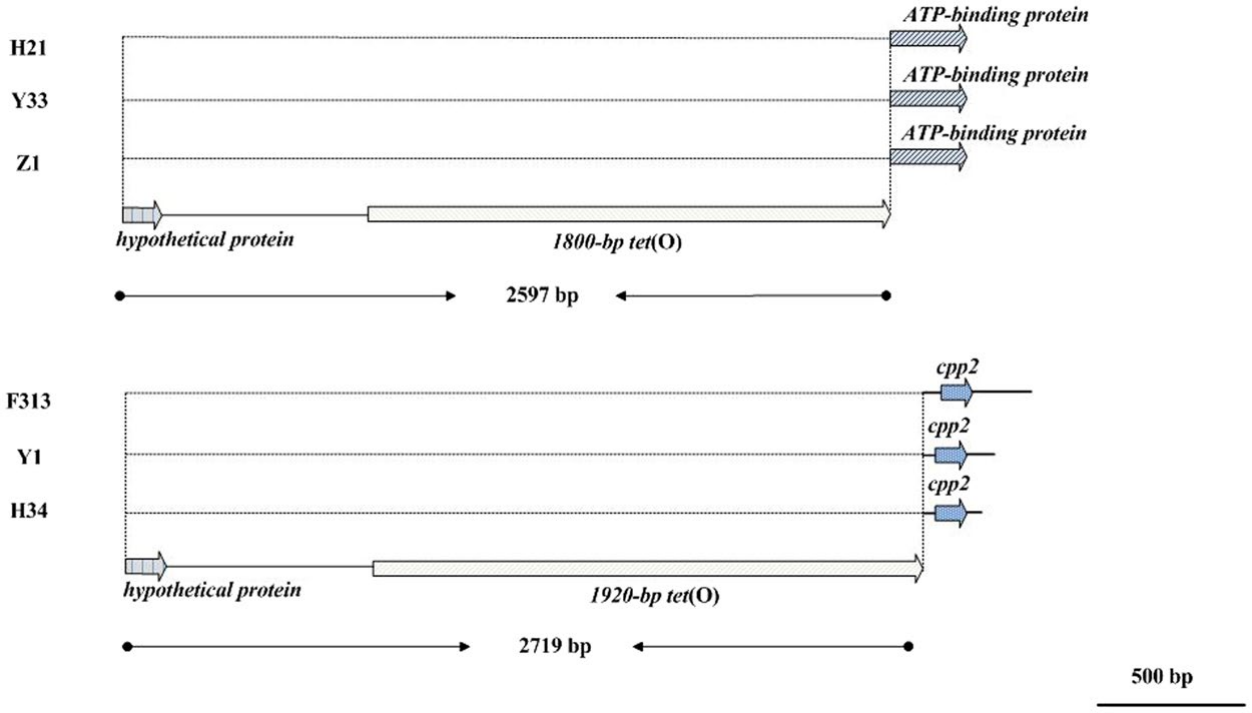
Genetic organization of the regions upstream and downstream of *tet*(O) in the 6 *tet*(O)-positive bifidobacterial strains.

**Figure 3:**
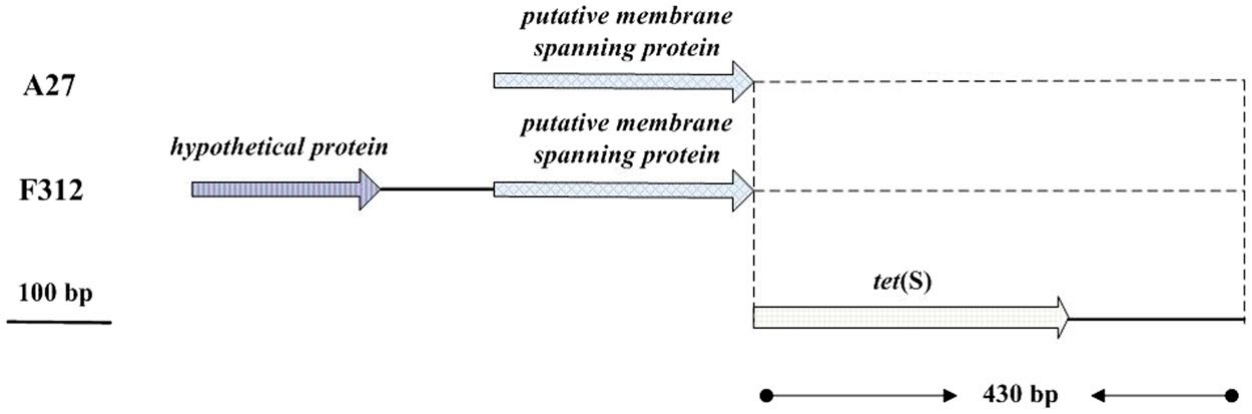
Genetic organization of the regions upstream and downstream of *tet*(S) in the 2 *tet*(S)-positive bifidobacterial strains.

The nucleotide sequences of the 1560-bp *te*t(W), 1457-bp *tet*(O), and 210-bp *tet*(S) genes and their flanking sequences were compared in different bifidobacterial strains (Figs 1, 2, 3).

The 21 *tet*(W)-positive bifidobacterial strains shared a core DNA region of 2281 bp, including a sequence of 298 bp, an upstream flanking sequence of 45 bp encoding an 14-amino-acid *tet*(W)-regulatory peptide, and the complete sequence of the 1920-bp *tet*(W) gene (Fig. 1). The 2281-bp sequence showed 99-100% nucleotide identity with the sequence previously identified in *Corynebacterium diphtheria* strain BH8 (GenBank CP003209.1), *Streptococcus suis* strain GZ1 (GenBank CP000837.1), and *Arcanobacterium pyogenes* strain OX4 (GenBank DQ517519.1).

Of the 6 *tet*(O)-positive bifidobacterial strains, three *B*. *longum* strains (H21, Y33 and Z1) shared a core DNA region of 2597 bp; however, an additional two *B*. *longum* strains, H34 and F313, and one *B*. *pseudocatenulatum* strain, Y1, shared a core DNA region of 2719 bp (Fig. 2). The conserved 2597-bp or 2719-bp sequences in the 6 *tet*(O)-positive bifidobacterial strains contained a sequence of 156 bp encoding a hypothetical protein, an upstream flanking sequence of 643 bp, and an 1800-bp or 1920-bp *tet*(O) gene, and exhibited 99-100% nucleotide identity with the 2597-bp or 2719-bp sequences previously identified in *Campylobacter coli* strain 6461 (GenBank JQ613156.1), *Streptococcus pyogenes* strain ICESp2905 (GenBank FR691055.1), and *Streptococcus suis* strain NSUI002 (GenBank CP011419.1).

The 2 *tet*(S)-positive bifidobacterial strains shared a core DNA region of 430 bp, including the 252-bp *tet*(S) gene and a downstream flanking sequence of 178 bp, which exhibited 99-100% identity with the sequences previously identified in *Listeria monocytogenes* strain LM78 (GenBank JX865374.1), *Streptococcus suis* strain G52 (GenBank JQ762256.1), and *Enterococcus faecium* strain E241 (GenBank JN980096.1).

### Analysis of ORFs in regions flanking the tet(W), tet(O), and tet(S) genes

In the 21 *tet*(W)-positive bifidobacterial strains shown in Fig. 1, a 23SrRNA gene was found 97 bp downstream of the *tet*(W) gene and showed 100% nucleotide identity with the sequence previously identified in *Bifidobacterium animalis* strain *A6* (GenBank CP010433.1). Additionally, another two open reading frames (ORFs), including a 140-bp sequence encoding thymidylate synthase and a 648-bp sequence encoding dihydrofolate reductase, were found upstream of the *tet*(W) gene in one *B*. *animalis subsp*. *lactis* strain, F12, which exhibited 98–100% nucleotide identity with the sequence previously identified in *Bifidobacterium pseudocatenulatum* DSM 20438 (GenBank AP012330.1) and *Bifidobacterium kashiwanohens*e PV20-2 (GenBank CP007456.1).

In the 6 *tet*(O)-positive bifidobacterial strains shown in Fig. 2, a 198-bp ORF encoding an ABC transporter was found downstream of the 1800-bp *tet*(O) gene in *B*. *longum* strains H21, Y33 and Z1, and a 99-bp cpp2 gene was found downstream of the 1920-bp *tet*(O) gene in *B*. *longum* strains H34 and F313 and *B*. *pseudocatenulatum* strain Y1.

In the 2 *tet*(S)-positive bifidobacterial strains (*B*. *pseudocatenulatum* strain F312 and *B*. *breve* strain A27), a 270-bp ORF encoding a putative membrane-spanning protein was found in the adjacent upstream region flanking the *tet*(S) gene. Additionally, in *B*. *pseudocatenulatum* strain F312, another 186-bp ORF encoding a hypothetical protein was found 400 bp upstream of the *tet*(S) gene.

### Mobility of the tet(W), tet(O), and tet(S) genes

Filter matings of the 21 *tet*(W)-positive bifidobacterial strains, the six *tet*(O)-positive bifidobacterial strains, and the two *tet*(S)-positive bifidobacterial strains with *Enterococcus faecalis* StF-EFM failed in laboratory conditions.

## Discussion

In our previous investigation of a collection of 92 bifidobacterial strains originating from the human GIT, the macrolide, lincosamide, and streptogramin (MLS) resistance gene *erm*(X) was detected in 30 bifidobacterial strains. This study further investigated the tetracycline-resistant phenotype and genotype of these 92 strains and found that 29 bifidobacterial strains exhibited tetracycline resistance. Notably, nine bifidobacterial strains, including *B*. *longum* strains F313 and F21, *B*. *pseudocatenulatum* strains J56, H23, Z25, a39, Y1, and F312, and *B*. *bifidum* strain Y21, simultaneously exhibited MLS and tetracycline resistance. Bifidobacteria have been regarded as traditional safe probiotics in the human GIT^7, 8^, and only *tet*(W)- and *tet*(M)-mediated tetracycline resistance had been reported in human intestinal bifidobacteria^10–13^. However in the present study, acquired tetracycline resistance in the 29 bifidobacterial strains was mediated by *tet*(W), *tet*(O) or *tet*(S), and this study provides the first report of *tet*(O)- and *tet*(S)-mediated tetracycline resistance in bifidobacteria. The finding of two new tetracycline resistance genes [*tet*(O) and *tet*(S)] in bifidobacteria suggest that the selective pressure of intensive tetracycline use has caused human intestinal bifidobacteria to acquire more tetracycline resistance genes to survive and eventually become a reservoir of tetracycline resistance genes as previously speculated by many researchers^17–19^.

It has been generally considered that the AR resistance genes carried by bifidobacteria are transferred from other bacteria in the human GIT via a number of complex mechanisms^15, 20^. Previously, it was reported that the tetracycline resistance gene *tet*(W) in 10 human intestinal bifidobacterial strains of 5 species had a conserved sequence of 2154 bp^10^. In the present study, the tetracycline resistance gene *tet*(W) in 21 human intestinal bifidobacterial strains of 4 species had a conserved sequence of 2281 bp that included the previously reported 2154 bp sequence, while the 1800-bp *tet*(O) gene in three human intestinal *B*. *longum* strains had a conserved sequence of 2599 bp, the 1920-bp *tet*(O) gene in another three human intestinal bifidobacterial strains of two species had a conserved sequence of 2719 bp, and the *tet*(S) gene in two human intestinal bifidobacterial strains of 2 species had a conserved sequence of 430 bp. All of these conserved sequences contained the sequence of the tetracycline resistance gene [*tet*(W), *tet*(O) or *tet*(S)] and its partial flanking sequence, which showed 98–100% nucleotide identity with the sequence previously identified in multiple human intestinal pathogens (*Arcanobacterium*, *Streptococcus*, *Corynebacterium*, *Campylobacter*, *Listeria*, etc.). Not unexpectedly, with the widespread use of tetracycline in the treatment of various human bacterial infections, pathogens are indeed more likely to harbor and retain AR genes and retain them than other bacteria in the human GIT^3, 21^. Therefore, our results indicate that different tetracycline resistance genes acquired by human intestinal bifidobacteria might initially be transferred from intestinal pathogens.

Because bifidobacteria rarely harbor plasmids, it is generally believed that the acquired AR genes tend to be integrated into the chromosome of bifidobacteria^22, 23^. However, the integration characteristics of the tetracycline resistance genes in the chromosome of human intestinal bifidobacteria are poorly understood. Previously, only one report had investigated the insertion site of the tetracycline resistance gene *tet*(W) in six intestinal bifidobacterial strains, showing that the *tet*(W) gene was flanked downstream by an *orf*Y gene in one *B*. *thermophilum* strain and one *B*. *longum* strain and by a transposase gene in two *B*. *longum* strains^12^. In the present study, the tetracycline resistance gene *tet*(W) was flanked downstream by a 23 S rRNA gene in 21 bifidobacterial strains, while the *tet*(S) was flanked upstream by a gene encoding a membrane-spanning protein in two bifidobacterial strains. In addition, in the six *tet*(O)-positive bifidobacterial strains, the *tet*(O) gene exhibited two different lengths, 1801 bp and 1920 bp; the 1800-bp *tet*(O) gene was flanked downstream by a gene encoding an ATP transporter, and the 1920-bp *tet*(O) gene was flanked downstream by a gene encoding a Cpp2 protein. Moreover, these genes flanking the *tet*(W), *tet*(O) or *tet*(S) in the bifidobacterial strains in this study only exhibited 98–100% nucleotide identity with these sequences previously identified in *Bifidobacterium*. Hence, our results provide evidence for revealing the insertion regularity of different tetracycline resistance genes into the chromosome of human intestinal bifidobacteria, and we speculate that each kind of acquired tetracycline resistance gene might tend to insert into the vicinity of specific genes in bifidobacteria. In Gram-positive anaerobes other than bifidobacteria, a few researchers had also investigated the integration characteristic of the acquired tetracycline resistance genes *tet*(W) and *tet*(S). However, no similar genes was found flanking the tetracycline resistance genes *tet*(W) in the two *Lactobacillus reuteri* strains^24^ and no similar genes were found flanking the tetracycline resistance genes *tet*(S) in the six *Streptococcus dysgalactiae* subsp. *equisimilis* strains^25^. Thus, unlike in bifidobacteria, the tetracycline resistance genes *tet*(W) and *tet*(S) in the other Gram-positive anaerobes might exhibit random insertion sites, which remains to be further studied.

Commercially used bifidobacterial strains are commonly screened from the healthy human GIT^26, 27^. However, it had been verified that one *B*. *longum* strain F8 isolated from the healthy human GIT could transfer the tetracycline resistance gene *tet*(W) to *Butyrivibrio adolescentis* strain L2-3229^12^. Thus, considering that the AR genes harbored by bifidobacterial strains could have the potential risk of transfer to pathogenic bacteria in the human GIT and become a treat to human healthy^28, 29^, the EFSA recommended that bacterial strains for commercial use should not harbor any transferable AR genes^16^. Over the past few years, only *tet*(W)- and *tet*(M)-mediated tetracycline resistance had been detected in human intestinal bifidobacteria^10–13^; thus, human intestinal bifidobacterial strains lacking the *tet*(W) and *tet*(M) genes would be considered as relatively safe. However, this study detected two new tetracycline resistance genes, *tet*(O) and *tet*(S), in human intestinal bifidobacteria in addition to *tet*(W) and further investigated the potential transferability of *tet*(W), *tet*(O) and *tet*(S) in bifidobacteria via filter mating experiments. Although no transfer of *tet*(W), *tet*(O) or *tet*(S) was observed via filter mating, this does not confirm that the *tet*(W), *tet*(O) or *tet*(S) in these bifidobacterial strains could not be transferred in the human GIT, since the actual transfer process of AR genes that occurs in the GIT usually occurs over a much longer period of time^15^. Therefore, the presence of the tetracycline resistance genes *tet*(O) and *tet*(S) should also be considered in the safety assessment of human intestinal bifidobacterial strains prior to commercial use.

In summary, this study has provided additional genetic knowledge regarding acquired tetracycline resistance in bifidobacteria isolated from the healthy human GIT. The detection of two new tetracycline resistance genes [*tet*(O) and *tet*(S)] in human bifidobacteria indicates that human intestinal bifidobacteria have begun to harbor more AR genes, and that the screening of bifidobacterial strains from the healthy human GIT for commercial use faces additional challenges.

## Methods

### Ethical Statement

Ethics approval for this study was obtained within the framework of the National Basic Research Program of China (973 Program) (No. 2012CB720802). Final approval was obtained from the Research Ethics Committee of Shanghai Jiaotong University, China. The methods were carried out in accordance with the approved guidelines. The written informed consent was obtained from all participants or their legal guardians in the study.

### Bacterial strains and growth conditions

One hundred individual bifidobacterial strains belonging to seven species were investigated in the present study: of these, one was a type strain, seven were commercial strains, and 92 were isolated from the feces of 14 healthy individuals (Tables 1 and 2). The first letter in the names of the 92 strains, “J”, “L”, “F”, “W”, “N”, “Y”, “A”, “Z”, “D”, “X”, “H”, “a”, “B”, or “S”, indicates the origin among the 14 individuals. The number of strains of each species in the 100 tested strains was as follows: *Bifidobacterium longum*, 45; *Bifidobacterium breve*, 18; *Bifidobacterium lactis* 19; *Bifidobacterium pseudocatenulatum*, 12; *Bifidobacterium bifidum*, 3; *Bifidobacterium adolescentis*, 2; *Bifidobacterium infantis*, 1.

All of the strains were cultured in de Man Rogosa Sharpe (MRS) medium supplemented with 0.05% (*w/v*) L-cysteine (MRSC). Incubations were performed at 37 °C for 12–48 h under anaerobic conditions (AnaeroGen^TM^, Oxoid Ltd, Basingstoke, UK).

### Antimicrobial susceptibility

The MIC values of tetracycline in these 100 bifidobacterial strains were determined using Etest strips (bioMérieux, Marcy-l’Étoile, France), according to the manufacturer’s recommendations. Prior to the assay, the strains were anaerobically cultured in MRSC medium at 37 °C for 24 h. An inoculum was then suspended in MRSC broth to achieve the turbidity of a 1.0 McFarland standard (3 × 10^8^ cells/ml) and was subsequently uniformly applied to an agar plate with a sterile cotton swab in three directions. After drying for 20 or 30 min, tetracycline Etest strips with antimicrobial gradients ranging from 0.016 to 256 μg/ml were placed on the agar plates. The MIC values were visually defined as the lowest tetracycline concentration at which no growth was observed with the Etest strip after aerobic incubation at 37 °C for 48 h. The interpretation of the tetracycline susceptibility status of these strains was based on the tetracycline breakpoint for Bifidobacterium (MIC = 8 μg/ml) defined by the EFSA^16^. Each assay was repeated three times in duplicate.

### PCR amplification and sequencing

Genomic DNA from the 100 bifidobacterial strains was extracted according to the method of Ausubel and colleagues^30^. The primers used to amplify five ribosomal protection genes [*tet*(M), *tet*(O), *tet*(S), *tet*(W), and *tet*(T)] and eight efflux genes [*tet*(A), *tet*(B), *tet*(C), *tet*(D), *tet*(E), *tet*(G), *tet*(K), and *tet*(L)] are listed in Table 4. The primers used to detect *tet*(M), *tet*(T), *tet*(A), *tet*(B), *tet*(C), *tet*(D), *tet*(E), *tet*(G), *tet*(K), and *tet*(L) were chosen as previously described^31–33^, while three sets of primers (tetW_F and tetW_R, tetO_F and tetO_R, and tetS_F and tetS_R) were designed to detect the *tet*(W), *tet*(O), and *tet*(S) genes based on the *tet*(W) sequence of *Bifidobacterium animalis subsp*. *lactis* CNCM I-2494 (GenBank CP002915.1), the *tet*(O) sequence of *Streptococcus suis* BM407 (GenBank FM252032.1), and the *tet*(S) sequence of *Lactococcus lactis subsp*. *lactis* strain ILIBB-JZK (GenBank KF278750.1), respectively. PCR assay was performed with TaKaRa Ex Taq DNA polymerase using the component concentration recommended by the provider (TaKaRa, Dalian, China). PCR products were separated by electrophoresis on a 1.0% agarose gel and visualized by ethidium bromide staining. All positive amplicons were purified by a PCR purification spin kit (Qiagen, Germany) and subsequently sequenced by the BGI Company (Shanghai, China). The obtained sequences were compared with those in GenBank.

**Table 4:** Primers used in the present study.

Name	Sequence (5′-3′)	Target	Reference
tetM_F	ACAGAAAGCTTATTATATAAC	*tet*(M)	32
tetM_R	TGGCGTGTCTATGATGTTCAC		
tetO_F	AACTTAGGCATTCTGGCTCAC	*tet*(O)	This study
tetO_R	CTATGGACAACCCGACAGAAG		
tetS_F	TAGATACTCCTGGACACAT	*tet*(S)	This study
tetS_R	ATGAGAATGACCTCGTTAC		
tetW_F	CGGATTGTGGCATTTGT	*tet*(W)	This study
tetW_R	GCATAGAGGGTGAAGGAG		
tetT_F	AAGGTTTATTATATAAAAGTG	*tet*(T)	34
tetT_R	AGGTGTATCTATGATATTTAC		
tetA_F	GTAATTCTGAGCACTGTCGC	*tet*(A)	32
tetA_R	CTGCCTGGACAACATTGCTT		
tetB_F	AAAACTTATTATATTATAGTG	*tet*(B)	34
tetB_R	TGGAGTATCAATAATATTCAC		
tetC_F	TCTAACAATGCGCTCATCGT	*tet*(C)	32
tetC_R	CGTTGAAGGCTCTCAAGGGC		
tetD_F	ATTACACTGCTGGACGCGAT	*tet*(D)	32
tetD_R	CTGATCAGCAGACAGATTGC		
tetE_F	GTGATGATGGCACTGGTCAT	*tet*(E)	32
tetE_R	CTCTGCTGTACATCGCTCTT		
tetG_F	TTTCGGATTCTTACGGTC	*tet*(G)	32
tetG_R	TCCTGCGATAGAGCTTAGA		
tetK_F	TTATGGTGGTTGTAGCTAGAAA	*tet*(K)	33
tetK_R	AAAGGGTTAGAAACTCTTGAAA		
tetL_F	GTMGTTGCGCGCTATATTCC	*tet*(L)	33
tetL_R	GTGAAMGRWAGCCCACCTAA		
tetW_U_SP1	GGAGGTTGTTTCCGCTTTGCTG	Upstream region of *tet*(W)	This study
tetW_U_SP2	GGTAAAGGAACCCACCGTCATT		
tetW_U_SP3	TCTGTTACACCACTCCCGCTTG		
tetW_D_SP1	CATCTGTGCCACTGGAAGGAAG	Downstream region of *tet*(W)	This study
tetW_D_SP2	TCCGTCCTCGTTGTCCCTTTTT		
tetW_D_SP3	AAGGTCGTCTTTCCAGCGTCTA		
tetO_U_SP1	GCAAATCAATCCCCTCTTGGTCA	Upstream region of *tet*(O)	This study
tetO_U_SP2	GTCTGTGCCTGTATGCCATCCTTT		
tetO_U_SP3	CCACTGAAAAGATGTCACTGCTGT		
tetO_D1_SP1	CGATACAGCCTGCTCTGGTGAT	Downstream region of the1457-bp *tet*(O)	This study
tetO_D1_SP2	CTCCCTATGCTCCAAACAACGA		
tetO_D1_SP3	TATTGCTTGGGGCACTTACAGA		
tetO_D2_SP1	TTTCTGGGCTTCTGTCGGGTTGTC	Downstream region of the 1800-bp *tet*(O)	This study
tetO_D2_SP2	AAATGCGGTTATGGAGGGGGTTCT		
tetO_D2_SP3	GCAGGGACAGAACTATTAGAGCCA		
tetS_U_SP1	GATAGCGGTACAACGAAAACGGTA	Upstream region of *tet*(S)	This study
tetS_U_SP2	TTTGGAACGCCAGAGAGGTATT		
tetS_U_SP3	CTGGACATGGATTTTTGGCAG		
tetS_D_SP1	TGCCAAAATCCATGGTCCAGG	Downstream region of *tet*(S)	This study
tetS_D_SP2	CGGTCTGAATAGTAATACCTGTGTGG		
tetS_D_SP3	CCGTTTTGGTTGTACCGCTATC		

### Genome walking

Nested PCR was conducted to amplify the flanking sequences of the *tet*(W) genes in 21 bifidobacterial strains, the *tet*(O) genes in 6 bifidobacterial strains, and the *tet*(S) genes in two bifidobacterial strains using a Genome Walking Kit (TaKaRa, Dalian, China), following the manufacturer’s recommendations. The nested PCR assays were performed in three steps using the same AP primer and three reverse SP primers (SP1, SP2, and SP3) designed under the conditions suggested by the kit instructions. The SP primers groups (SP1, SP2, and SP3) are listed in Table 3 and were designed to amplify the upstream and downstream sequences flanking the *tet*(W), *tet*(S), and *tet*(O) genes. In particular, two groups of SP primers were designed to amplify the downstream flanking sequences of the 1457-bp and 1800-bp *tet*(O) genes. All positive amplicons obtained in the third cycle of nested PCR were purified by a PCR purification spin kit (Qiagen, Germany) and subsequently sequenced by the BGI Company (Shanghai, China).

### Filter mating experiments

The potential transferability of the *tet*(W) genes from 21 bifidobacterial strains, the *tet*(O) genes from 6 bifidobacterial strains, and the *tet*(S) genes from two bifidobacterial strains (donors) to *Enterococcus faecalis* StF-EFM (recipient) was investigated by filter mating experiments, following the method of Gevers and colleagues^34^. Briefly, the donor and recipient cells were grown to mid-exponential phase in MRSC medium prior to assay, and 1 ml of donor and 1 ml of recipient culture were mixed. Subsequently, the mixture (2 ml) was dispensed onto a sterile filter (0.45 μm; MF-Millipore membrane filter, HAWP 02500, Millipore) that was then anaerobically incubated on non-selective BHI agar (Oxoid) at 37 °C for 24 h. The cells were collected from the filters by centrifugation and resuspended in 1 ml of PBS. The transconjugants were aerobically detected on Pfizer Enterococcus Selective (PSE) agar supplemented with tetracycline (16 μg/ml), since only *Enterococcus faecalis* StF-EFM (recipient) can grow on PSE agar under aerobic conditions. Transfer frequencies were defined as the number of transconjugant colonies per recipient colony formed after the mating period.

### Nucleotide sequence accession numbers

The nucleotide sequences of the regions flanking the *tet*(W) gene in 21 bifidobacterial strains were submitted to the GenBank database under accession numbers KY682293-KY682303, KY689744-KY689752, and KY697297. The nucleotide sequences of the regions flanking the *tet*(O) gene in 6 bifidobacterial strains were submitted to the GenBank database under accession numbers KY697298-KY697303. The nucleotide sequences of the regions flanking the *tet*(S) gene in the 2 bifidobacterial strains were submitted in the GenBank database under accession numbers KY818315 and KY818316.

### Data Availability

The datasets generated during the current study are included in this article and are available from the corresponding author on reasonable request.
